# Ribavirin-Induced Anemia in Hepatitis C Virus Patients Undergoing Combination Therapy

**DOI:** 10.1371/journal.pcbi.1001072

**Published:** 2011-02-03

**Authors:** Sheeja M. Krishnan, Narendra M. Dixit

**Affiliations:** 1 Department of Chemical Engineering, Indian Institute of Science, Bangalore, India; 2 Bioinformatics Centre, Indian Institute of Science, Bangalore, India; Emory University, United States of America

## Abstract

The current standard of care for hepatitis C virus (HCV) infection – combination therapy with pegylated interferon and ribavirin – elicits sustained responses in only ∼50% of the patients treated. No alternatives exist for patients who do not respond to combination therapy. Addition of ribavirin substantially improves response rates to interferon and lowers relapse rates following the cessation of therapy, suggesting that increasing ribavirin exposure may further improve treatment response. A key limitation, however, is the toxic side-effect of ribavirin, hemolytic anemia, which often necessitates a reduction of ribavirin dosage and compromises treatment response. Maximizing treatment response thus requires striking a balance between the antiviral and hemolytic activities of ribavirin. Current models of viral kinetics describe the enhancement of treatment response due to ribavirin. Ribavirin-induced anemia, however, remains poorly understood and precludes rational optimization of combination therapy. Here, we develop a new mathematical model of the population dynamics of erythrocytes that quantitatively describes ribavirin-induced anemia in HCV patients. Based on the assumption that ribavirin accumulation decreases erythrocyte lifespan in a dose-dependent manner, model predictions capture several independent experimental observations of the accumulation of ribavirin in erythrocytes and the resulting decline of hemoglobin in HCV patients undergoing combination therapy, estimate the reduced erythrocyte lifespan during therapy, and describe inter-patient variations in the severity of ribavirin-induced anemia. Further, model predictions estimate the threshold ribavirin exposure beyond which anemia becomes intolerable and suggest guidelines for the usage of growth hormones, such as erythropoietin, that stimulate erythrocyte production and avert the reduction of ribavirin dosage, thereby improving treatment response. Our model thus facilitates, in conjunction with models of viral kinetics, the rational identification of treatment protocols that maximize treatment response while curtailing side effects.

## Author Summary

The treatment of HCV infection poses a major global health-care challenge today. The current standard of care, combination therapy with interferon and ribavirin, works in only about half of the patients treated. Because no alternatives are available yet for patients in whom combination therapy fails, identifying ways to improve response to combination therapy is critical. Increasing exposure to ribavirin does improve response but is associated with the severe side-effect, anemia. One way to maximize treatment response therefore is to increase ribavirin exposure to levels just below where anemia becomes intolerable. A second way is to supplement combination therapy with growth hormones, such as erythropoietin, that increase the production of red blood cells (erythrocytes) and compensate for ribavirin-induced anemia. Rational optimization of combination therapy thus relies on a quantitative description of ribavirin-induced anemia, which is currently lacking. Here, we develop a model of the population dynamics of erythrocytes in individuals exposed to ribavirin that quantitatively describes ribavirin-induced anemia. Model predictions capture several independent observations of ribavirin-induced anemia in HCV patients undergoing combination therapy, estimate the threshold ribavirin exposure beyond which anemia becomes intolerable, suggest guidelines for the usage of growth hormones, and facilitate rational optimization of therapy.

## Introduction

130–170 million people worldwide are currently infected with hepatitis C virus (HCV) [1]. Over 70% of HCV infections become chronic and if untreated may lead to cirrhosis and hepatocellular carcinoma, necessitating liver transplantation [1]. The standard of care for HCV infection involves combination therapy with pegylated interferon and ribavirin [2]. Ribavirin alone does not elicit a lasting antiviral response [3]–[6], yet it substantially improves treatment response in combination with interferon [7]–[11]. For instance, whereas ∼29% of the patients treated with interferon exhibited a sustained virological response (SVR), the response rate increased to ∼56% upon addition of ribavirin [8]. Ribavirin, however, is associated with the side-effect, hemolytic anemia, which often renders therapy intolerable [4], [12]–[15]. With the standard ribavirin dosage of 1000–1200 mg/day, 54% of the patients treated experienced a decline in the hemoglobin (Hb) level of over 3 g/dL, and 10% of the men and 7% of the women treated experienced an Hb decline of over 5 g/dL (normal Hb range: 14–16 g/dL) [15]. This drop in Hb often necessitates a reduction of ribavirin dosage, which significantly compromises treatment response [7], [13], [14], [16]. The probability of achieving SVR is estimated to decrease from ∼65% to ∼45% when ribavirin dosage is reduced from ∼15 mg/kg to ∼7 mg/kg of body weight, in combination with pegylated interferon at 1.5 µg/kg of body weight [7]. Patients receiving fewer than 60% of the planned ribavirin doses had lower response rates [14], indicating that lower cumulative ribavirin exposure results in poorer treatment response [13], [16]. The rates of relapse of infection following the end of treatment also increased upon lowering ribavirin dosage [14], [16]. In a recent clinical trial where interferon was employed with telaprevir, a promising new inhibitor of HCV protease, response rates were lowest in patients who were not administered ribavirin [17], underscoring the importance of ribavirin in achieving SVR.

Alternatives for patients who do not respond to combination therapy do not exist yet [2], [18]. Significant efforts are underway therefore to identify treatment protocols that maximize response rates to combination therapy while curtailing side-effects [16], [19]–[25]. A particularly promising strategy is to supplement combination therapy with growth hormones, such as erythropoietin, that stimulate erythropoiesis and thus avert the reduction of ribavirin dosage, potentially improving treatment response [26]–[31]. The predominant mechanism(s) of the anti-HCV activity of ribavirin remain to be established [32]–[34]. Mathematical models of viral kinetics have been developed that describe the antiviral activity of interferon and the enhancement of treatment response rates due to ribavirin, and are being extended to predict the impact of new antiviral drugs [34]–[42]. Ribavirin-induced anemia, on the other hand, remains poorly understood [13], [24], [25], [43]–[47] and precludes rational optimization of combination therapy.

Here, we construct a mathematical model of the population dynamics of erythrocytes that quantitatively describes ribavirin-induced anemia and informs future strategies for improving outcomes of combination therapy. Model predictions capture experimental observations of the accumulation of ribavirin in erythrocytes and the ensuing Hb decline in HCV patients following the onset of combination therapy, estimate the enhanced turnover rate of erythrocytes during therapy and the threshold ribavirin exposure beyond which anemia is intolerable, present guidelines for the optimal usage of growth hormone supplements, and provide a framework, in conjunction with models of viral kinetics, for rational optimization of combination therapy.

## Results

### Model formulation

**Figure 1:**
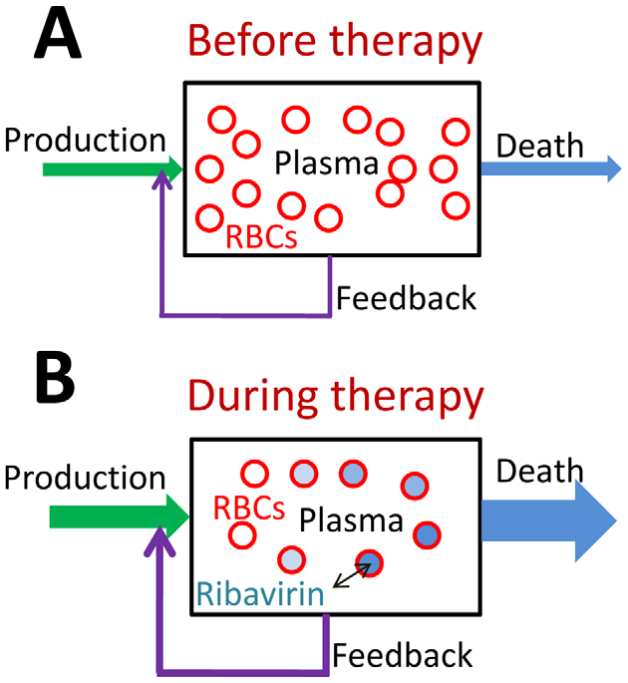
Schematic of the population dynamics of RBCs in HCV infected individuals. **A**, Before the onset of combination therapy, the production and death rates of RBCs are balanced and the population of RBCs in plasma is constant. **B**, Following the onset of therapy, ribavirin enters RBCs and increases their death rates. Cells born at different times are exposed to ribavirin for different durations and hence carry different concentrations of ribavirin. The enhanced death rate lowers the RBC population, which triggers an increase in the RBC production from the bone marrow by a negative feedback through the hormone erythropoietin.

Prior to the onset of treatment with ribavirin, the population of erythrocytes (RBCs) in an HCV infected individual is constant; a balance exists between RBC production and death (Fig. 1). Following the onset of treatment, ribavirin administered orally gets rapidly transported from the plasma to RBCs, where it is phosphorylated to its mono-, di- and tri-phosphate analogs (RMP, RDP, and RTP) [48]. Phosphorylated analogs are neither easily metabolized nor transported out of RBCs [48]. Consequently, ribavirin accumulates inside RBCs in the form of its phosphorylated analogs; the total intracellular concentration of ribavirin can be >100-fold its extracellular concentration [47]. This dramatic accumulation of ribavirin may induce oxidative damage and result in enhanced extra vascular death of RBCs [12]. Indeed, RBC lifespan decreased from 107±22 d in HCV patients not exposed to ribavirin to 39±13 d in HCV patients undergoing treatment with ribavirin [49], [50]. The shortened RBC lifespan creates an imbalance between RBC production and death and results in a decline in the RBC population. Accordingly, Hb levels drop and patients become anemic. We construct a mathematical model to describe this dynamics of ribavirin-induced anemia (Methods).

### Model predictions

#### Population dynamics of RBCs during treatment with ribavirin

**Figure 2:**
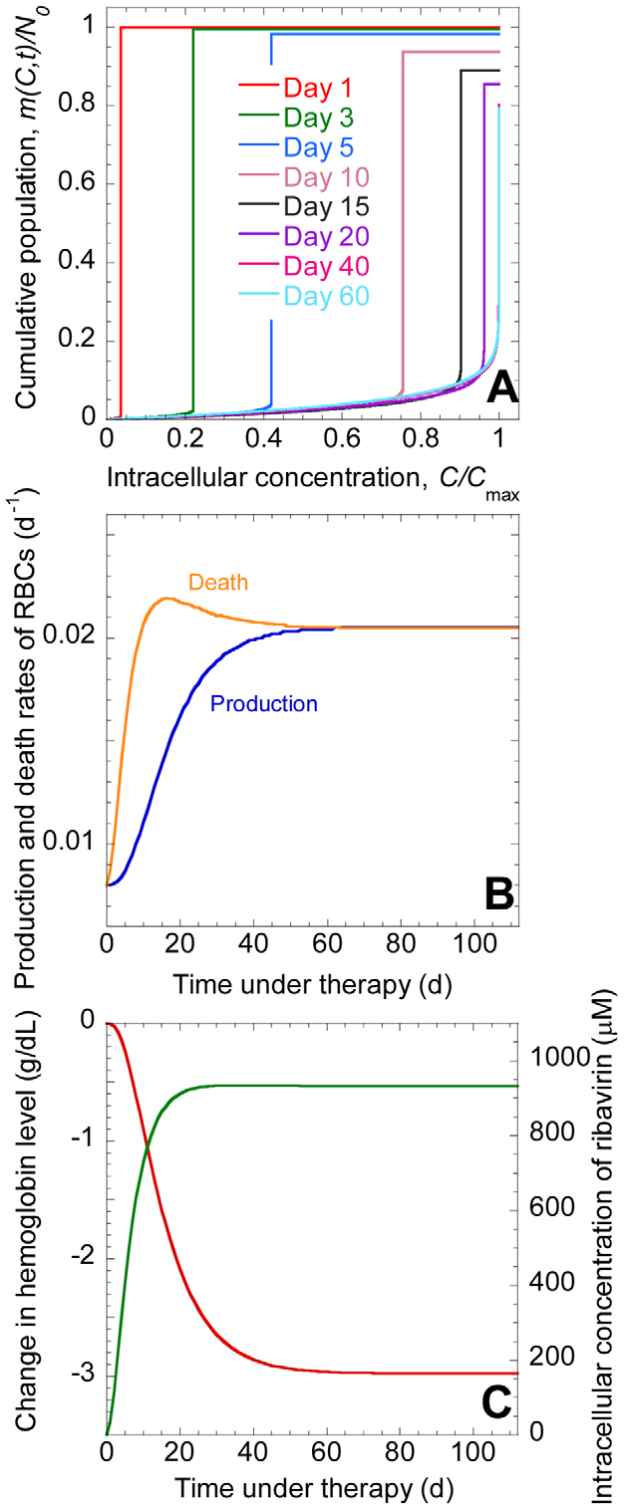
Model predictions of the dynamics of ribavirin-induced anemia. **A**, The cumulative population, *m*(*C*,*t*), of RBCs as a function of the intracellular concentration of RXP, *C*, at different times, *t*, following the onset of combination therapy, predicted by Eqs. (1)–(3). Parameters employed are listed in Table 1. *m*(*C*,*t*) is normalized by *N*_0_ and *C* by *C*_max_ = 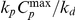. **B**, The production rate, *P*(*t*), and the death rate 
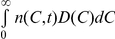
 of RBCs. **C**, The corresponding change in *Hb* (red) and *C*_avg_ (green).

We present model predictions in terms of the cumulative population, 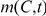, which is the population of RBCs at time *t* following the onset of treatment in which the concentration of ribavirin phosphorylated analogs, RXP, is less than or equal to *C* (Fig. 2A). (RXP comprises RMP, RDP, and RTP.) At the start of treatment 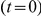, no cells contain RXP, and 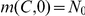 for all *C*, where *N*_0_ is the steady population of RBCs prior to the onset of treatment. In other words, the population of cells carrying RXP at concentrations smaller than or equal to *C* is *N*_0_ for all 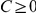. At 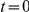, the production and death rates of RBCs are in balance (Fig. 2B), the hemoglobin level, *Hb* = *Hb*_0_, and the average intracellular concentration of ribavirin, 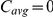 (Fig. 2C). With time, ribavirin accumulates inside cells. At any time 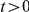, a distribution of RXP concentrations across cells emerges with cells exposed to ribavirin longer possessing higher concentrations of RXP. Thus, cells present from the start of treatment possess the highest concentration of RXP. At 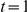 d, for instance, the latter cells possess RXP at the concentration 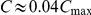, where *C*_max_ = 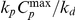 is the maximum intracellular concentration of RXP for a given extracellular concentration of ribavirin 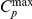 (Fig. 2A). Cells born after the onset of treatment possess RXP at concentrations smaller than 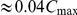 at 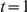 d. Because most of the cells present at 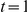 d are from the population that existed at the start of therapy, 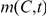 exhibits a sharp rise at 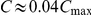 and reaches the value *N*(*t* = 1) (Fig. 2A). With time, the sharp rise in 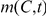 shifts to higher values of 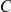 (Fig. 2A) indicating greater accumulation of RXP. Accordingly, 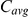 increases (Fig. 2C). Cells die at increasing rates as intracellular RXP accumulates (Fig. 2B). The population of RBCs, *N*(*t*), and, hence, 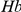 correspondingly decrease (Fig. 2C). New cells are continuously produced at a rate that increases as the RBC population declines (Fig. 2B). Eventually, a new balance between RBC production and death is attained, 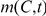 reaches a steady distribution, 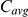 reaches an asymptotic maximum 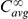, and 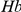 attains a new, lower steady value 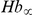.

#### Factors that influence the severity of ribavirin-induced anemia

**Figure 3:**
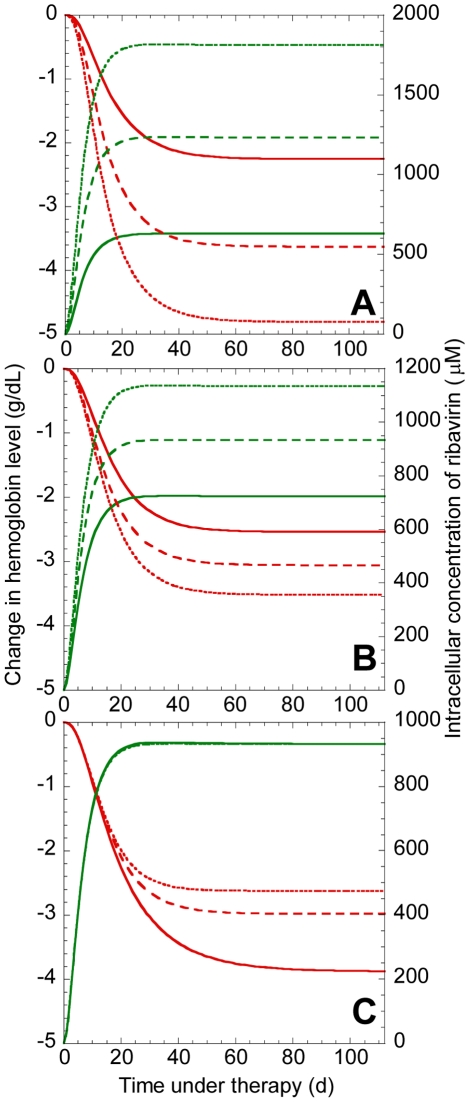
Factors influencing the dynamics of ribavirin-induced anemia. Changes in *Hb* (red) and *C*_avg_ (green) predicted by Eqs. (1)–(3) for different parameter values: **A**, 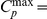
5 µM (solid lines), 10 µM (dashed lines) and 15 µM (dotted lines). **B**, 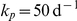 (solid lines), 65 d^−1^ (dashed lines), 80 d^−1^ (dotted lines). **C**, 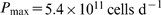 (solid lines), 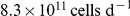 (dashed lines), 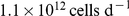 (dotted lines). The other parameters are mentioned in Table 1.

An increase in the steady state plasma concentration of ribavirin, 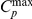, which may be achieved with a higher dosage, results in an increase in 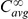 and a decrease in 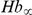, illustrating the more severe anemia that results with increased ribavirin exposure. For instance, increasing 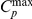 from 5 µM to 15 µM increases the total drop in *Hb*, 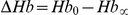, from ∼2 g/dL to ∼5 g/dL (Fig. 3A). An increase in the intracellular phosphorylation rate, 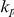, (or a decrease in the loss rate of intracellular RXP, 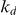) results in a higher 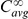 for the same 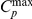, which in turn elevates cell death rates and lowers 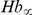. Thus, 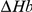 increases from ∼2.5 g/dL when 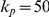 d^−1^ to ∼3.5 g/dL when 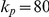 d^−1^ (Fig. 3B), illustrating that differences in the intracellular metabolism of ribavirin may contribute to inter-patient variations in the severity of ribavirin-induced anemia. Enhancing the RBC production rate, by increasing 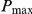 (see Eq. (3)), (or lowering the sensitivity of the RBC death rate to ribavirin accumulation, by increasing 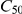 or decreasing 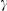 (see Eq. (2))), reduces 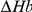 (Fig. 3C). Administration of growth hormones, such as erythropoietin, enhances the RBC production rate, which reduces 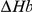 for the same ribavirin exposure and thus improves the tolerability of ribavirin. Similarly, decrease in the intracellular inosine triphosphatase level, observed recently in some patients [51], may interfere with RTP activity and lower the sensitivity of the RBC death rate to ribavirin, which also reduces 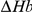 and improves the tolerability of ribavirin.

#### Inter-patient variations

**Figure 4:**
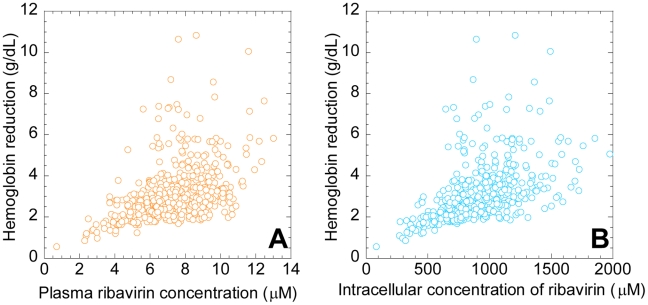
Model predictions of the variations in the severity of ribavirin-induced anemia. The reduction in *Hb* (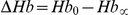) predicted by Eqs. (1)–(3) as a function of **A**, 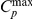, and **B**, 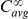, for 500 different combinations of the parameter values (mean±s.d.) 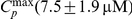, 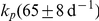, 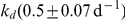, 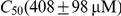, and 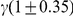. The standard deviations on the parameter values correspond to the 95% confidence limits obtained from the best-fits to patient data (see Fig. 5). The other parameters are mentioned in Table 1.

Inter-patient variability in the severity of anemia may arise from variations in the intracellular uptake, accumulation, or metabolism of ribavirin, as well as in the dependence of the RBC lifespan on ribavirin accumulation and in the sensitivity of the RBC production rate to changes in *Hb*. To obtain a measure of this inter-patient variability, we calculated 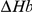 using 500 different combinations of the values of the parameters, 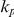, 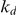, 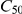 and 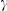, for a range of values of 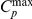. The parameter values for each combination were chosen randomly from Gaussian distributions based on the mean values and confidence levels of the respective parameters obtained from comparisons of our model predictions with patient data (see below). Indeed, we find that inter-patient variations in 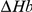 may be substantial for any ribavirin exposure (Fig. 4A) or intracellular accumulation (Fig. 4B). Further, the variation increases with increase in ribavirin exposure and intracellular accumulation.

Below, we compare our model predictions with experiments.

### Comparisons of model predictions with patient data

**Figure 5:**
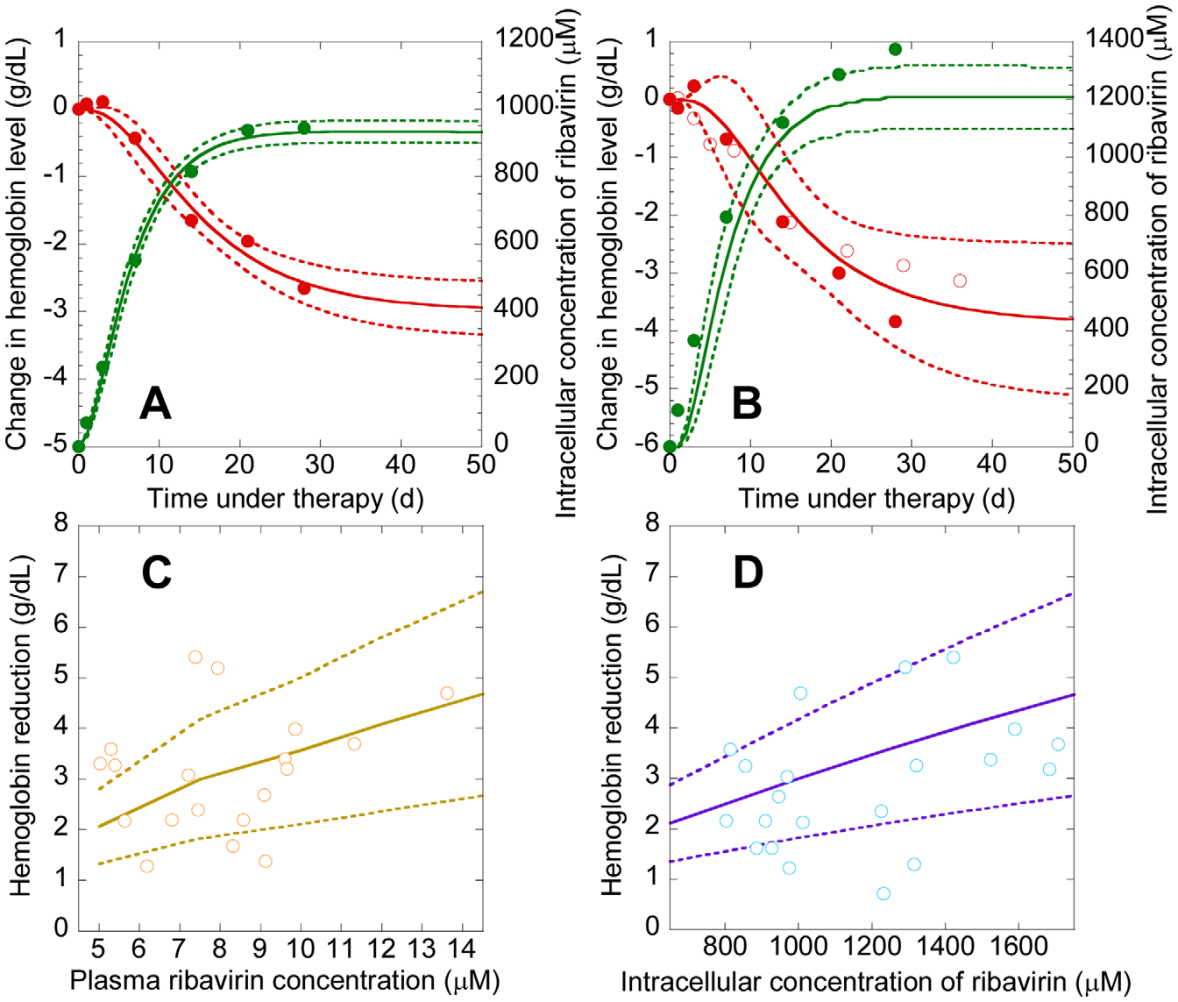
Comparisons of model predictions with experiments. **A**, Best-fits of model predictions (lines) of *Hb* (red) and *C*_avg_ (green) with experimental data (symbols) from 7 Japanese patients with 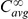
<1000 µM [47]. We let *Hb*_0_ = 14.4 g/dL and 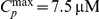 following the mean reported values for these patients [47]. We use 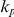, 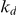, 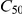, and 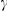 as adjustable parameters. The remaining parameters are mentioned in Table 1. The resulting best-fit parameter estimates (95% CI) are 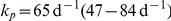, 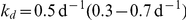, 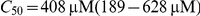, and 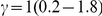. Dashed lines show 95% confidence intervals on the predictions. **B**, Comparisons of our predictions (lines) of *Hb* (red) and *C*_avg_ (green) using the parameters above with data (symbols) from 12 Japanese patients with 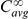
>1000 µM (solid circles) [47]. In the latter patients, the mean *Hb*_0_≈15 g/dL and 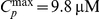. We also show comparisons with independent data from 20 patients [29] (open circles) with mean *Hb*_0_ = 15.1 g/dL. Solid lines are predictions with *Hb*_0_ = 15.1 g/dL and 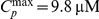, and the mean parameters mentioned above, and dashed lines represent standard deviations. **C**, **D**, Model predictions (lines) and experimental observations (symbols) of the reduction in *Hb* (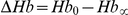) under combination therapy as a function of **C**, 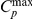, and **D**, 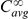, in 19 Japanese patients [47]. Solid lines represent predictions with the mean parameter values above and dashed lines represent standard deviations obtained from several hundred realizations of our model predictions for different combinations of the values of 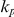, 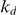, 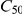, and 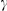 generated randomly from distributions based on the best-fit parameter estimates and 95% confidence limits mentioned above.

We consider a recent study of the time-evolution of 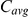 and 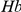 in 19 Japanese patients following the onset of combination therapy [47]. In this latter study, no reduction of ribavirin dosage is reported. The patients were divided into two groups based on whether 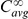
<1000 µM (7 patients) or 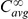
>1000 µM (12 patients); the data are reported as the average within each group. We fit model predictions of 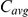 and 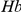 to the data of the former 7 patients using 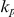, 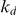, 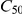 and 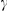 as adjustable parameters. (Interferon may also induce anemia, but does so to a much smaller extent than ribavirin [13]. We therefore assume that the *Hb* decline in patients undergoing combination therapy is primarily due to ribavirin.) We fix the remaining parameters based on previous studies or from analysis of independent experiments (Methods). Model predictions provide good fits to the data and yield estimates of 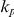, 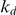, 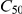 and 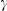 (Fig. 5A). The fits suggest that our model is able to describe the underlying dynamics of ribavirin-induced anemia in HCV patients.

Interestingly, with the same parameter values, our model captures changes in *Hb* and 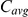 from the other 12 Japanese patients, as well as an independent data set of *Hb* decline in another group of HCV patients undergoing combination therapy [29] (Fig. 5B), validating our best-fit parameter estimates. Further, with the same parameter values, we estimate that the RBC lifespan is 38 days (95% CI: 19–55 days) in Japanese patients with 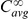
<1000 µM and 33 days (95% CI: 14–53 days) in Japanese patients with 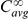
>1000 µM. These estimates of the RBC lifespan are in close agreement with independent estimates, 39±13 days, from measurements of alveolar carbon monoxide [49], [50], presenting another successful test of our model. Finally, we find that our predictions of the dependence of 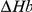 on 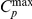 and 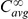 using the same parameters above are also in agreement with observations in the Japanese patients [47] (Fig. 5C,D). Our model thus presents a robust description of ribavirin-induced anemia in HCV patients undergoing combination therapy.

### Clinical implications

**Figure 6:**
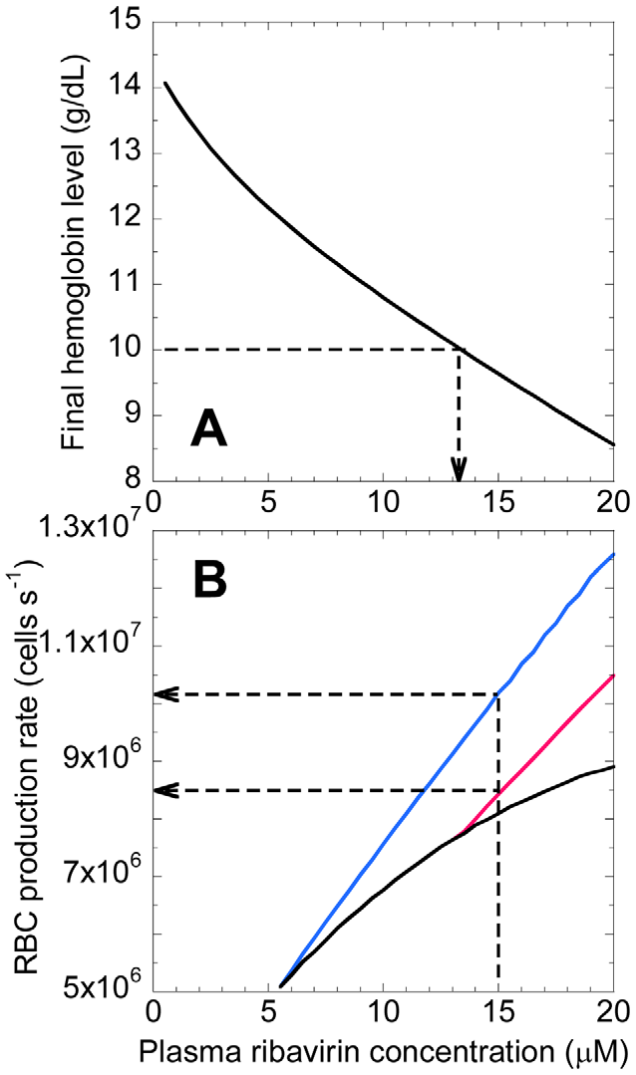
Model predictions of threshold ribavirin exposure and requisite RBC production. **A**, Prediction of 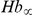 as a function of 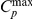. The arrow indicates the threshold 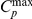 above which 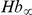
<10 g/dL. **B**, Predictions of the RBC production rate during therapy (black) and the production rate required to maintain 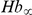 of 10 g/dL (pink) and 12 g/dL (blue) as functions of 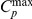. The arrows indicate the desired production rates when 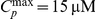. Parameter values employed are mentioned in Table 1.

Our model has several clinical implications. First, it enables estimation of the threshold ribavirin exposure beyond which anemia is intolerable. Current treatment guidelines recommend a reduction of ribavirin dosage when *Hb* decreases below 10 g/dL. We apply our model to predict 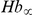 as a function of 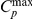. We find that on average (when 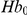 = 14.4 g/dL) 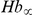
<10 g/dL when 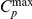
>13 µM (Fig. 6A). Thus, steady state plasma concentrations above 13 µM would render ribavirin therapy intolerable. While the dependence of the peak plasma concentration on dosage following a single ribavirin dose has been determined [48], the dependence of 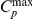 on dosage remains to be established. A description of the multiple dose pharmacokinetics of ribavirin, which also remains elusive [6], [34], [48], [52], [53], would establish the dosage corresponding to 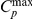 of 13 µM that would render ribavirin intolerable.

Second, when 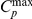 is above the threshold, our model allows estimation of the increase in RBC production, which may be achieved by administration of exogenous growth hormones such as recombinant erythropoietin, necessary to avert the currently recommended reduction of dosage. Because growth hormones also have side-effects [29], [54], one strategy is to use them at levels just enough to increase 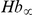 to 10–12 g/dL (rather than the pretreatment level), which renders ribavirin tolerable [16]. We apply our model to predict the level of RBC production necessary for achieving 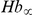 of 10–12 g/dL for different values of 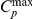 (Fig. 6B). Thus, when 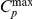 = 15 µM, RBC production rates of 8.44 and 10.2 million cells s^−1^ are necessary for ensuring 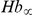 of 10 and 12 g/dL, respectively. Increase in endogenous erythropoietin levels during therapy, also observed experimentally [45], [55], results in an enhanced production rate of 8.1 million cells s^−1^, which is 3.5-fold higher than the basal production rate (here 2.3 million cells s^−1^ in the absence of ribavirin) but inadequate to achieve the desired 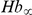. Hormone supplements may be employed to provide the balance of 0.34 or 2.1 million cells s^−1^ increase in the RBC production rate to ensure 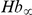 of 10 or 12 g/dL, respectively. This deficiency in RBC production that hormone supplements must compensate increases with ribavirin exposure (Fig. 6B).

## Discussion

The ability to enhance treatment response rates renders ribavirin central to the treatment of HCV infection. Maximizing the benefit of ribavirin to patients requires striking the right balance between its antiviral activity and its treatment-limiting side-effect, hemolytic anemia. Rational approaches to therapy optimization thus rely on quantitative descriptions of both the antiviral and the hemolytic activities of ribavirin. Extant mathematical models predict the enhancement in treatment response due to ribavirin [34]–[42]. Ribavirin-induced anemia, however, remains poorly described and limits our ability to maximize treatment response. Here, we fill this gap by constructing a model of the population dynamics of RBCs that quantitatively describes ribavirin-induced anemia. By assuming that intracellular accumulation of ribavirin enhances RBC death rate in a dose-dependent manner, our model captures several independent observations of ribavirin-induced anemia in HCV patients undergoing combination therapy. In particular, our model predicts the dynamics of the accumulation of ribavirin in RBCs and the resulting decline of *Hb* in patients following the onset of therapy, estimates the reduced lifespan of RBCs during therapy, and describes inter-patient variations in the severity of anemia, thus presenting a robust description of ribavirin-induced anemia, which, in conjunction with models of viral kinetics, may facilitate identification of treatment protocols that maximize the impact of ribavirin in the treatment of HCV infection.

Our model has clinical implications. First, it allows estimation of the threshold ribavirin exposure beyond which ribavirin-induced anemia becomes intolerable. For instance, with model parameters that describe ribavirin-induced anemia in the patients we considered (Fig. 5), we estimate that steady state plasma ribavirin concentrations above 13 µM would render ribavirin therapy intolerable. Determining dosage levels corresponding to this steady state plasma concentration requires knowledge of the pharmacokinetics of ribavirin, which is currently lacking [6], [34], [48], [52], [53]. Ribavirin pharmacokinetics is peculiar because of an unusually long elimination phase that follows rapid absorption and distribution phases upon oral dosing [48]. Standard absorption-elimination models of drug pharmacokinetics are unable to describe this long elimination phase. Models that include additional compartments have been proposed to capture the three-phase pharmacokinetics of ribavirin [52], but the biological origin of these compartments remains unclear. An additional complication is that the half-life of the elimination phase increases from 79 h following a single dose to 274–298 h following multiple dosing [48], suggesting that parameters that describe single dose pharmacokinetics may not apply to multiple dose pharmacokinetics. In the absence of rigorous models of ribavirin pharmacokinetics, one may have to rely on empirical relationships between the dosage and the resulting steady state plasma concentration following multiple dosing (e.g., [56]) to establish the dosage that would ensure tolerability of ribavirin while maximizing treatment response.

Second, our model suggests guidelines for the usage of hormone supplements, such as erythropoietin, which enhance RBC production and improve the tolerability of ribavirin. For instance, we predict that when ribavirin accumulates to a plasma concentration of 15 µM, the associated enhanced RBC death rate elicits a natural response that increases RBC production 3.5-fold, from 2.3 to 8.1 million cells s^−1^. This response, however, is inadequate to suppress ribavirin-induced anemia adequately and renders ribavirin intolerable. We estimate then that growth hormone supplements must increase RBC production rate by an additional 0.34–2.1 million cells s^−1^ to render ribavirin tolerable. This compensation that hormone supplements must provide increases with ribavirin accumulation. Identifying the dosage of the growth hormones that induces the necessary RBC production requires knowledge of the dose-response relationships and of the pharmacokinetics of the growth hormones, which are yet to be fully elucidated [26]–[31].

Third, genetic variations that resulted in a deficiency in the enzyme inosine triphosphatase (ITPA) were recently found to protect HCV patients against ribavirin-induced anemia [51]. Deficiency in ITPA causes an increase in inosine triphosphate levels in RBCs, which is thought to interfere with RTP activity and thereby suppress the hemolytic potential of ribavirin. Because deficiency in ITPA is a clinically benign condition, therapeutic intervention to suppress ITPA presents a promising new strategy to curtail ribavirin-induced anemia without compromising the antiviral activity of ribavirin [51]. Our model may be adapted to inform the development of such an intervention strategy. In our model, the dependence of the death rate of RBCs on ribavirin accumulation, determined by Eq. (2) (Methods), would now be a function of the ITPA level. Thus, experiments that determine how variations in the ITPA level both in the absence and in the presence of ribavirin influence RBC lifespan would provide the necessary inputs for our model to account explicitly for the role of ITPA in ribavirin-induced anemia. The resulting model would enable determination of the minimal inhibition of ITPA necessary to maintain ribavirin-induced anemia within tolerable limits. Conversely, using information of the ITPA level intrinsic to a patient, the model can be applied to predict the maximum ribavirin dosage that the patient can tolerate, thus presenting an avenue for personalizing the treatment of HCV infection.

## Methods

### Model development

We consider the RBC population in an individual at time *t* following the onset of treatment with ribavirin (*t* = 0) (Fig. 1). RBCs produced at different times in the interval from 0 to *t* will have been exposed to ribavirin for different durations and accordingly have different intracellular levels of ribavirin. We define 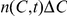 as the population of RBCs that contain ribavirin phosphorylated analogs, RXP, which comprises RMP, RDP, and RTP, at concentrations between 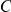 and 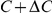 at time 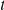. 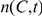 is thus the number density of RBCs containing RXP at concentration *C* at time *t*. The time evolution of 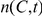 is governed by the following equation (Text S1)
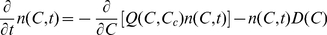
(1)


The first term on the right-hand-side in Eq. (1) represents the change in 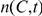 due to intracellular phosphorylation of ribavirin. 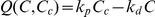 is the net rate of increase of *C* due to phosphorylation, 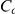 is the intracellular concentration of (unphosphorylated) ribavirin, 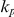 is the phosphorylation rate and 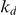 is the rate of loss, including by possible slow dephosphorylation, of RXP. In vitro studies of ribavirin uptake by RBCs observe rapid (<10 min) equilibration of intracellular and extracellular ribavirin [53], [57]. We assume therefore that 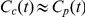, the concentration of ribavirin in plasma. With twice daily oral administration of ribavirin, 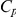 rises from zero at 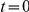 and reaches an asymptotic maximum, 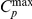, so that 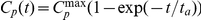, where 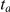 is the characteristic timescale of the accumulation of ribavirin in plasma [6], [38].

The second term on the right-hand side of Eq. (1) accounts for the loss of RBCs due to their death. We assume that the death rate, *D*, of RBCs increases with 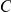 as follows
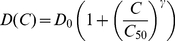
(2)where 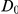 is the death rate of RBCs in the absence of ribavirin, 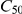 is that value of 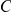 at which the death rate doubles (or the lifespan halves) compared to that in the absence of ribavirin, and 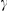, analogous to the Hill coefficient, determines the sensitivity of 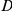 to changes in 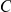. (A saturable form for *D*(*C*) appears inconsistent with available data; see Text S2, Fig. S1.)

Equation (1) is constrained by the initial condition that 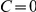 in all cells at the start of therapy, so that 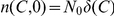, where *N*_0_ is the population of RBCs at *t* = 0, and 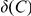 is the Dirac delta function, which satisfies 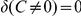 and 
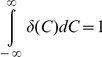
. In other words, the Dirac delta function ensures that no cells have RXP at non-zero concentrations at *t* = 0. A second constraint on Eq. (1) is imposed by the boundary condition that when 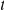
>0, newborn cells contain no RXP so that 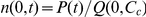 (Text S1) where
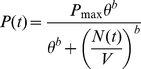
(3)is the rate of production of RBCs at time *t*.

The production of RBCs by the bone marrow is regulated by a negative feedback mechanism involving the hormone erythropoietin [58]. Recent studies on modeling erythropoiesis elucidate the complexities involved in a quantitative description of this feedback mechanism [59]–[65]. Here, we employ Eq. (3) to capture the essential features of this negative feedback: As the population of RBCs, 
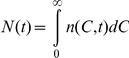
, decreases, *P* increases. 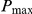 is the maximum production rate of RBCs, which occurs when *N* is vanishingly small, 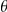 is that value of the RBC population per unit volume of blood (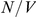) at which 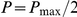, 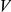 is the volume of blood, and 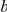, analogous to the Hill coefficient, determines the sensitivity of 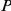 to changes in 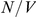. Eq. (3) provides good fits to independent measurements of the dynamics of the recovery of RBCs following phlebotomy (Text S3, Fig. S2).

Equations (1)–(3) present a model of the population dynamics of RBCs in individuals undergoing treatment with ribavirin. We solve the equations (see below) and obtain the population density, 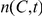, and the corresponding cumulative population, 
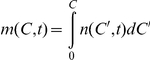
, using which we predict the time-evolution of the hemoglobin level in blood, 
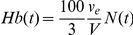
 (where 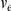 is the volume of a single erythrocyte); the average concentration of ribavirin in RBCs, 
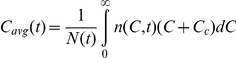
; and the average RBC lifespan, 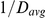, where 
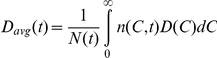
 is the average death rate of RBCs.

### Solution of model equations using the method of characteristics

Equation (1) along with the initial and boundary conditions is equivalent to the following set of differential equations obtained using the method of characteristics (Text S4)
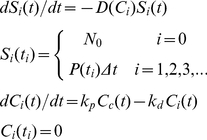
(4)where *S_i_*(*t*) is the subpopulation of cells born within an interval 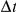 of 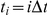 that survive at time *t*. *C_i_*(*t*) is the concentration of RXP in the latter cells at time *t*. We solve Eq. (4) along with Eqs. (2) and (3) with 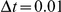 d using a program written in MATLAB (Text S5). We validate our solution methodology against an analytical solution that can be obtained in the limiting case when the RBC death rate is independent of RXP accumulation (Text S6, Fig. S3). We also ensure that 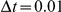 d allows accurate integration of Eq. (4) without compromising computational efficiency (Fig. S4). From the solution, we calculate the quantities of interest, viz., 
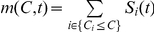
, 
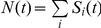
, 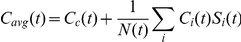, and 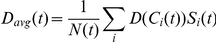.

### Model parameters

We employ the following values of the model parameters unless stated otherwise. The average RBC lifespan in normal man is ∼120 days [49], [66], which corresponds to 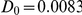 d^−1^. We let *b* = 7 following earlier studies [59] and obtain 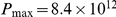 cells d^−1^ from an independent analysis of blood loss experiments (Text S3). We fix 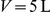 and 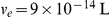 [67]. Using 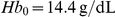 [47], we get 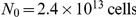. We obtain 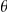 from the initial steady state 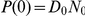. Further, we let 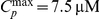 [47] and because ribavirin accumulates in plasma to its maximum concentration in ∼4 weeks, we set 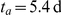 [6], [38]. The remaining parameter values 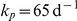, 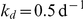, 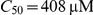, and 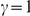 are obtained from best-fits of our model predictions to experimental data (Fig. 5A). We summarize model parameters and their values in Table 1.

**Table 1:** Summary of model parameters and their values employed.

Parameter	Description	Value (95% CI)^a^	Source
*V*	Volume of blood	5 L	[67]
*v_e_*	Volume of one RBC	9×10^−14^ L	[67]
*t_a_*	Characteristic timescale of accumulation of RBV in plasma	5.4 d	[6], [38]
*b*	Coefficient determining sensitivity of RBC production rate to changes in number per volume of RBCs	7	[59]
*D*_0_	Death rate of RBCs in the absence of ribavirin	8.3×10^−3^ d^−1^	[49], [66]
*Hb*_0_	Initial Hb level	14.4 g/dL	[47]
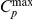	Steady state plasma RBV concentration	7.5 µM	[47]
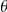	RBC population per unit volume at which RBC production rate is half maximal		Determined from pretreatment steady state, 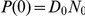
*P_max_*	Maximum production rate of RBCs	8.4×10^12^ cells d^−1^	Best-fit (Text S3)
*k_p_*	Phosphorylation rate of ribavirin in RBCs	65 (47–84) d^−1^	Best-fit (Fig. 5A)
*k_d_*	Loss rate of ribavirin phosphorylated analogs in RBCs	0.5 (0.3–0.7) d^−1^	Best-fit (Fig. 5A)
*C*_50_	Concentration of ribavirin phosphorylated analogs at which RBC death rate is twice the pretreatment value	408 (189–628) µM	Best-fit (Fig. 5A)
*γ*	Coefficient determining sensitivity of RBC death rate to changes in concentration of ribavirin phosphorylated analogs	1 (0.2–1.8)	Best-fit (Fig. 5A)

a Typical values employed. Variations are indicated in the text and in figure legends.

### Fits of model predictions to patient data

We fit model predictions to experimental data (Fig. 5A) using the nonlinear regression tool NLINFIT in MATLAB.

## Supporting Information

Figure S1 Hemoglobin reduction as a function of the intracellular ribavirin concentration.
(0.09 MB PDF)

Figure S2 Analysis of RBC recovery following phlebotomy.
(0.41 MB PDF)

Figure S3 Validation of the solution methodology.
(0.39 MB PDF)

Figure S4 Sensitivity of the numerical solution to the integration time step.
(0.37 MB PDF)

Text S1 Derivation of equation (1) and its boundary condition.
(0.29 MB PDF)

Text S2 Dependence of RBC death rate on intracellular ribavirin concentration.
(0.19 MB PDF)

Text S3 Analysis of phlebotomy experiments.
(0.28 MB PDF)

Text S4 Solution of model equations using the method of characteristics.
(0.30 MB PDF)

Text S5 MATLAB program for solving model equations.
(0.19 MB PDF)

Text S6 Validation of the solution methodology.
(0.20 MB PDF)
