# Supplementary material for: Ribavirin-Induced Anemia in Hepatitis C Virus Patients Undergoing Combination Therapy
Source: PLoS Comput Biol. 2011 Feb 3;7(2):e1001072. doi: 10.1371/journal.pcbi.1001072 (PMC3033369; doi:10.1371/journal.pcbi.1001072)
Supplement: Figure S1 — Hemoglobin reduction as a function of the intracellular ribavirin concentration. (0.09 MB PDF) [file pcbi.1001072.s001.pdf]

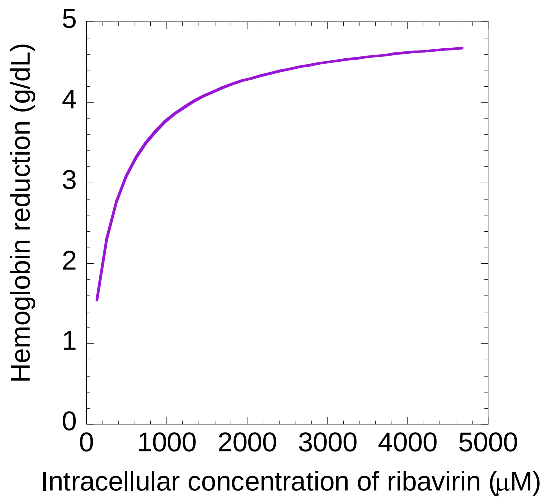

Figure S1. Hemoglobin reduction as a function of the intracellular ribavirin concentration. Model prediction (line) of the reduction in Hb ($\Delta Hb = Hb_0 - Hb_\infty$) as a function of C_{avg} calculated as in Fig. 5D but with Eq. (2) replaced by $D(C) = D_0 + (D_\mathrm{m} - D_0)\dfrac{C}{C + C_{50}}$ so that D reaches an asymptotic maximum of D_m for large values of C. Parameter values employed are $k_p = 65\,\mathrm{d}^{-1}$, $k_d = 0.5\,\mathrm{d}^{-1}$, $C_{50} = 400\,\mu\mathrm{M}$, and $D_m = 0.05\,\mathrm{d}^{-1}$. All the other parameter values are the same as in Fig. 2.
