# Supplementary material for: Ribavirin-Induced Anemia in Hepatitis C Virus Patients Undergoing Combination Therapy
Source: PLoS Comput Biol. 2011 Feb 3;7(2):e1001072. doi: 10.1371/journal.pcbi.1001072 (PMC3033369; doi:10.1371/journal.pcbi.1001072)
Supplement: Figure S2 — Analysis of RBC recovery following phlebotomy. (0.41 MB PDF) [file pcbi.1001072.s002.pdf]

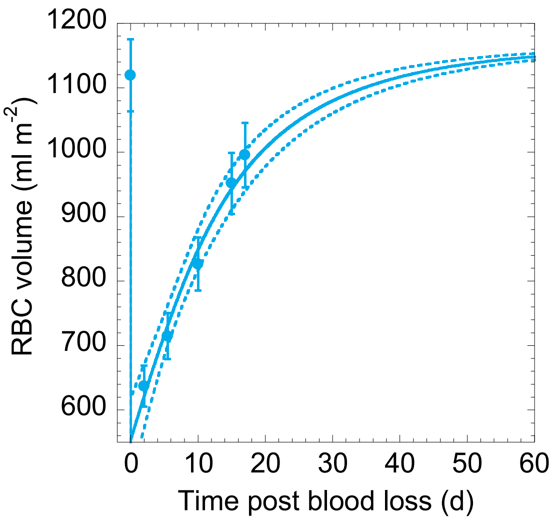

Figure S2. Analysis of RBC recovery following phlebotomy. Best-fit prediction (line) of the recovery of RBC volume per unit body surface area (median body surface area was 1.89 m^2) following phlebotomy using Eqs. (S3.1)-(S3.3) compared with experimental observations (symbols). We fix b=7 and obtain θ from Eq. (S3.3) (see Text S3). We also let $V = 5\,\mathrm{L}$, $v_e = 9\times10^{-14}\,\mathrm{L}$, and N_0 corresponding to an RBC volume of 2.2 L. N_{loss} and $P_{\max}$ are used as adjustable parameters. The resulting best-fit estimates are $P_{\max}$ = 8.3×10^{11} cells d^{-1} (95% CI: 5.3×10^{11} -1.0×10^{12} cells d^{-1}) and N_{loss} corresponding to RBC volume of 1.1 L (95% CI: 1.0-1.2 L). The error bars are maximum and minimum values of the measurements across patients. The dotted lines are 95% confidence intervals on the predictions.
