# Supplementary material for: Ribavirin-Induced Anemia in Hepatitis C Virus Patients Undergoing Combination Therapy
Source: PLoS Comput Biol. 2011 Feb 3;7(2):e1001072. doi: 10.1371/journal.pcbi.1001072 (PMC3033369; doi:10.1371/journal.pcbi.1001072)
Supplement: Figure S3 — Validation of the solution methodology. (0.39 MB PDF) [file pcbi.1001072.s003.pdf]

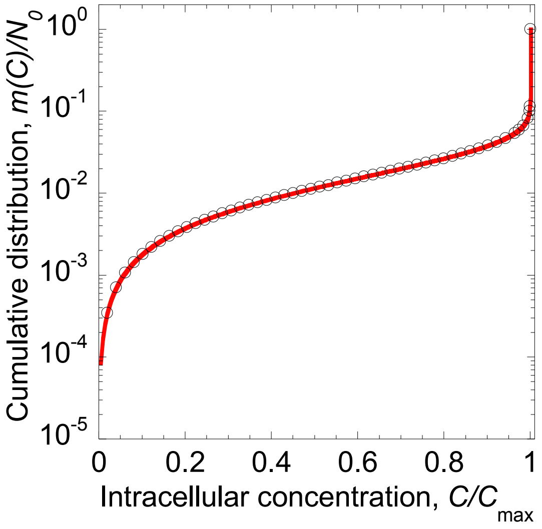

Figure S3. Validation of the solution methodology. Comparison of the numerical solution (symbols) obtained by integrating Eqs. (4) when $D(C_i)=D_0$ at steady state with the corresponding analytical solution (line) provided by Eq. (S6.4). Parameter values employed are listed in Table 1.
