# Supplementary material for: Ribavirin-Induced Anemia in Hepatitis C Virus Patients Undergoing Combination Therapy
Source: PLoS Comput Biol. 2011 Feb 3;7(2):e1001072. doi: 10.1371/journal.pcbi.1001072 (PMC3033369; doi:10.1371/journal.pcbi.1001072)
Supplement: Figure S4 — Sensitivity of the numerical solution to the integration time step. (0.37 MB PDF) [file pcbi.1001072.s004.pdf]

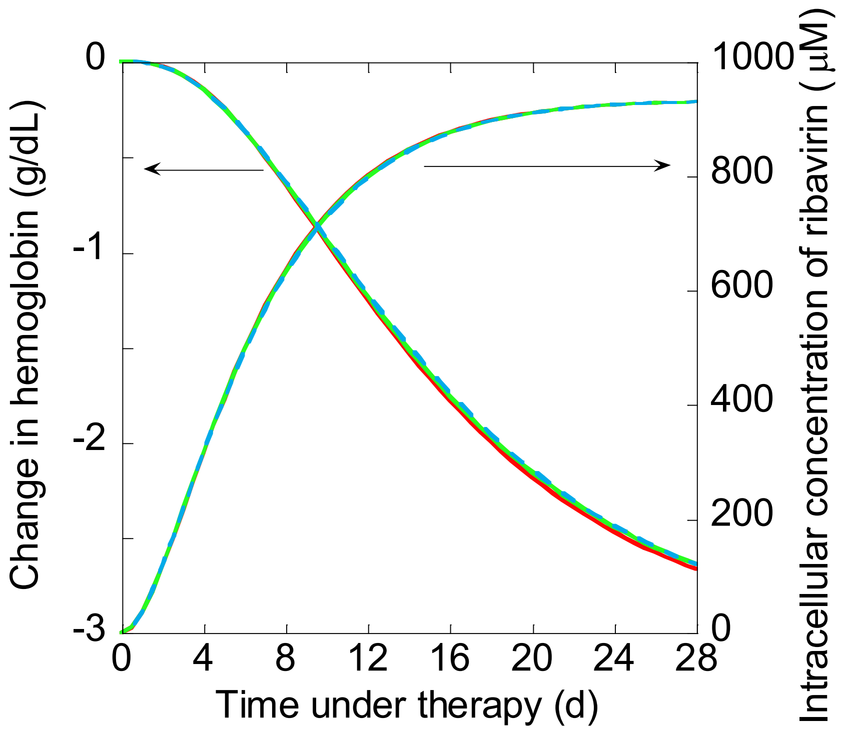

Figure S4. Sensitivity of the numerical solution to the integration time step. Comparison of the solution obtained by integrating Eq. (4) using three different values of the integration time step: $\Delta t = 0.02$ d (red), 0.01 d (green), and 0.005 d (blue). Parameter values employed are listed in Methods. The decline in *Hb* (left y axis) is identical for $\Delta t = 0.01$ d and 0.005 d and does not change for smaller values of Δt (not shown). The accumulation of intracellular ribavirin (right y axis) is identical for all the three values of Δt.
