# Supplementary material for: Ribavirin-Induced Anemia in Hepatitis C Virus Patients Undergoing Combination Therapy
Source: PLoS Comput Biol. 2011 Feb 3;7(2):e1001072. doi: 10.1371/journal.pcbi.1001072 (PMC3033369; doi:10.1371/journal.pcbi.1001072)
Supplement: Text S1 — Derivation of equation (1) and its boundary condition. (0.29 MB PDF) [file pcbi.1001072.s005.pdf]

Derivation of equation (1) and its boundary condition

To determine the time evolution of $n(C,t)$, we consider the fate of the cells $n(C,t)\Delta C$ an infinitesimally small time interval Δt later. In the interval Δt, an expected fraction $D(C)\Delta t$ of the cells die, where $D(C)$ is the death rate of RBCs in which the concentration of RXP is C. In the surviving population, $n(C,t)\Delta C\left(1-D(C)\Delta t\right)$, the intracellular concentration of RXP increases by the amount $Q(C,C_c)\Delta t$, where $Q(C,C_c)=k_pC_c-k_dC$ is the net rate of increase of C due to phosphorylation, C_c is the intracellular concentration of (unphosphorylated) ribavirin, k_p is the phosphorylation rate and k_d is the rate of loss, including by possible slow dephosphorylation, of RXP. Consequently, cells with RXP concentration within $Q(C,C_c)\Delta t$ of $C+\Delta C$ at time t will have RXP concentrations larger than $C+\Delta C$ at time $t+\Delta t$. Assuming that the RXP concentration is uniformly distributed in the range C to $C+\Delta C$ at time *t*, it follows that the fraction $Q(C,C_c)\Delta t/\Delta C$ of the surviving cells are lost due to the increased concentration of RXP. Consequently, the population $n(C,t)\Delta C\left(1-D(C)\Delta t\right)\left(1-Q(C,C_c)\Delta t/\Delta C\right)$ survives and continues to carry RXP at concentrations between C and $C+\Delta C$ at time $t+\Delta t$. Similarly, considering the fate of the cells $n(C-\Delta C,t)\Delta C$, it follows that the subpopulation $n(C-\Delta C,t)\Delta C\left(1-D(C-\Delta C)\Delta t\right)\left(Q(C-\Delta C,C_c)\Delta t/\Delta C\right)$ will survive at time $t+\Delta t$ and have RXP at concentrations between C and $C+\Delta C$. Thus, the population of cells at time $t+\Delta t$ with RXP at concentrations between C and $C+\Delta C$ would be $n(C,t+\Delta t)\Delta C=$

$$n(C,t)\Delta C\left(1-D(C)\Delta t\right)\left(1-Q(C,C_c)\frac{\Delta t}{\Delta C}\right)+n(C-\Delta C,t)\Delta C\left(1-D(C-\Delta C)\Delta t\right)\left(Q(C-\Delta C,C_c)\frac{\Delta t}{\Delta C}\right).$$

Rearranging terms, dividing by Δt and ΔC, and letting $\Delta t\to 0$ and $\Delta C\to 0$ yields Eq. (1):

$$\frac{\partial}{\partial t} n(C,t) = -\frac{\partial}{\partial C}[Q(C,C_c)n(C,t)] - n(C,t)D(C)$$

Equation (1) is constrained by the boundary condition that when t >0, newborn cells contain no RXP. Thus, at any time $t > 0$, the cells $n(0,t)\Delta C$, with RXP concentration between 0 and ΔC, would be the cells produced in the interval $\Delta C / Q(0,C_c)$ preceding t. Thus, $n(0,t)\Delta C = P(t)\Delta C / Q(0,C_c)$, or $n(0,t) = P(t) / Q(0,C_c)$, where $P(t)$ is the rate of production of RBCs at time t given by Eq. (3).
