# Supplementary material for: Ribavirin-Induced Anemia in Hepatitis C Virus Patients Undergoing Combination Therapy
Source: PLoS Comput Biol. 2011 Feb 3;7(2):e1001072. doi: 10.1371/journal.pcbi.1001072 (PMC3033369; doi:10.1371/journal.pcbi.1001072)
Supplement: Text S2 — Dependence of RBC death rate on intracellular ribavirin concentration. (0.19 MB PDF) [file pcbi.1001072.s006.pdf]

**Dependence of RBC death rate on intracellular ribavirin concentration**

We assume in Eq. (2) that the death rate, *D*, of RBCs increases with $C$. At large values of *C*, the death rate may become independent of *C* if other cellular processes and not the ribavirin concentration limit cell death, suggesting that *D* be a saturable function of *C* (such as a Hill-function). It follows then that beyond a certain value of $C_{avg}^{\infty}$, where *D* becomes independent of *C*, the total drop in *Hb*, $\Delta Hb = Hb_0 - Hb_{\infty}$, asymptotically reaches a plateau (Fig. S1). Available data (Fig. 5), however, show a significant positive correlation between $\Delta Hb$ and $C_{avg}^{\infty}$ ($R$=0.36, $P$<0.05) suggesting an increase of *D* with *C* in the range of values of *C* of interest here (800-1600 μM; Fig. 5), which is captured by the form of *D*(*C*) in Eq. (2).
