# Supplementary material for: Ribavirin-Induced Anemia in Hepatitis C Virus Patients Undergoing Combination Therapy
Source: PLoS Comput Biol. 2011 Feb 3;7(2):e1001072. doi: 10.1371/journal.pcbi.1001072 (PMC3033369; doi:10.1371/journal.pcbi.1001072)
Supplement: Text S3 — Analysis of phlebotomy experiments. (0.28 MB PDF) [file pcbi.1001072.s007.pdf]

Analysis of phlebotomy experiments

Alexanian *et al.* measured the recovery of RBC volume following blood loss [1]. In 9 healthy volunteers, 740-1300 ml of RBC volume was removed within 3 days and all plasma returned immediately. It was ensured that no significant change in the total blood volume occurred. RBC volume recovered after ~20 days following the blood loss (Fig. S2).

The dynamics of the RBC population in these patients may be described using the following equation

$$\frac{dN}{dt} = P - D_0 N \tag{S3.1}$$

where N is the population of RBCs at time t following blood loss, D_0 is the death rate of RBCs, and

$$P = \frac{P_{\max}\theta^b}{\theta^b + \left(\frac{N}{V}\right)^b} \tag{S3.2}$$

is the rate of RBC production, defined in Eq. (3). Our aim is to estimate the parameters in Eq. (S3.2) by analysis of the data of RBC recovery following blood loss. Before blood loss, the RBC population is constant at N_0, so that

$$\frac{P_{\max}\theta^b}{\theta^b + \left(\frac{N_0}{V}\right)^b} = D_0 N_0 \tag{S3.3}$$

which provides a constraint on the parameters values. We let b=7 following earlier studies [2]. We fit the evolution of the RBC volume, Nv_e, obtained by solving Eq. (S3.1) with the initial condition that just after blood loss (t=0), N=N_0-N_{loss}, where N_{loss} is the number of RBCs removed during phlebotomy. We use N_{loss} and $P_{\max}$ as adjustable parameters. Our model provides good fits to the data (Fig. S2) and yields $P_{\max} = 8.3\times10^{11}$ cells d^{-1} (95% CI: 5.3×10^{11} -1.0×10^{12} cells d^{-1})

and N_{loss} corresponding to an RBC volume of 1.1 L (95% CI: 1.0-1.2 L). Eq. (S3.3) then yields $\theta = 4.16 \times 10^{12}$ cells/L.

SUPPLEMENTAL REFERENCES

1. Alexanian R, Alfrey C (1970) Erythropoiesis in the anemia of bone marrow failure. J Clin Invest 49: 1986-1992.
2. Mahaffy JM, Belair J, Mackey MC (1998) Hematopoietic model with moving boundary condition and state dependent delay: applications in erythropoiesis. J Theor Biol 190: 135-146.
