# Supplementary material for: Ribavirin-Induced Anemia in Hepatitis C Virus Patients Undergoing Combination Therapy
Source: PLoS Comput Biol. 2011 Feb 3;7(2):e1001072. doi: 10.1371/journal.pcbi.1001072 (PMC3033369; doi:10.1371/journal.pcbi.1001072)
Supplement: Text S4 — Solution of model equations using the method of characteristics. (0.30 MB PDF) [file pcbi.1001072.s008.pdf]

Solution of model equations using the method of characteristics

To derive the solution technique, we integrate Eq. (1) with respect to C from $a_i(t)$ to $b_i(t)$:

$$\int_{a_i(t)}^{b_i(t)} \frac{\partial}{\partial t} n(C,t)dC = -\int_{a_i(t)}^{b_i(t)} \frac{\partial}{\partial C}(Q(C,C_c)n(C,t))dC - \int_{a_i(t)}^{b_i(t)} n(C,t)D(C)dC \qquad \text{(S4.1)}$$

Using Leibnitz rule to transform the integral on the left hand side and using the product rule to differentiate the integrand of the first term on the right hand side, we obtain

$$\begin{aligned}&\frac{d}{dt}\int_{a_i(t)}^{b_i(t)} n(C,t)dC - n(b_i(t),t)\frac{db_i(t)}{dt} + n(a_i(t),t)\frac{da_i(t)}{dt}\\ &= -\int_{a_i(t)}^{b_i(t)} n(C,t)\frac{d}{dC}Q(C,C_c)dC - \int_{a_i(t)}^{b_i(t)} Q(C,C_c)\frac{\partial}{\partial C}n(C,t)dC - \int_{a_i(t)}^{b_i(t)} n(C,t)D(C)dC\end{aligned} \qquad \text{(S4.2)}$$

Integrating the first term on the right hand side of Eq. (S4.2) by parts yields

$$\begin{aligned}&\frac{d}{dt}\int_{a_i(t)}^{b_i(t)} n(C,t)dC - n(b_i(t),t)\frac{db_i(t)}{dt} + n(a_i(t),t)\frac{da_i(t)}{dt}\\ &= -n(b_i(t),t)Q(b_i(t),C_c) + n(a_i(t),t)Q(a_i(t),C_c) - \int_{a_i(t)}^{b_i(t)} n(C,t)D(C)dC\end{aligned} \qquad \text{(S4.3)}$$

We now choose $a_i(t)$ and $b_i(t)$ to satisfy the differential equations:

$$\begin{aligned}\frac{da_i(t)}{dt} &= Q(a_i(t),C_c); \qquad a_i(t_i) = 0\\ \frac{db_i(t)}{dt} &= Q(b_i(t),C_c); \quad b_i(t_i) = Q(0,C_c)\Delta t\end{aligned} \qquad \text{(S4.4)}$$

This choice of $a_i(t)$ and $b_i(t)$ ensures the following. When $t=t_i$, $a_i(t_i) = 0$ and $b_i(t_i) = Q(0,C_c)\Delta t$, so that $\int_{a_i(t_i)}^{b_i(t_i)} n(C,t)dC$ comprises all cells with intracellular concentration of RXP between 0 and $Q(0,C_c)\Delta t$. In other words, $\int_{a_i(t_i)}^{b_i(t_i)} n(C,t)dC$ is the population of cells first exposed to ribavirin within an interval Δt of t_i. We denote this latter population by S_i. For

$t>t_i$, it follows from Eq. (S4.4) that the intracellular concentration of RXP in the population S_i lies within the range $a_i(t)$ to $b_i(t)$. Thus, $\int_{a_i(t)}^{b_i(t)} n(C,t)dC$ yields the size of the population S_i at time t. Using this definition of S_i and combining Eqs. (S4.3) and (S4.4), we obtain

$$\frac{dS_i}{dt} = -\int_{a_i(t)}^{b_i(t)} n(C,t)D(C)dC \qquad \text{(S4.5)}$$

Letting Δt be arbitrarily small so that $a_i(t) \approx b_i(t)$, which we denote by $C_i(t)$, (or using the mean value theorem) Eq. (S4.5) simplifies to

$$\frac{dS_i}{dt} = -D(C_i)S_i \qquad \text{(S4.6)}$$

Eq. (S4.6) thus governs the time evolution of S_i, the population of RBCs first exposed to ribavirin within an interval Δt of t_i. We choose $t_i = i\Delta t$, where $i = 0,1,2,...$ Thus, S_0 represents the population of RBCs at the onset of therapy (t=0) and S_1, S_2, etc., the populations of RBCs born respectively in the intervals 0 to Δt, Δt to $2\Delta t$, etc. We thus obtain the initial conditions for solving Eq. (S4.6):

$$\begin{aligned} S_0(0) &= N_0 \\ S_i(t_i) &= P(t_i)\Delta t \end{aligned} \qquad \text{(S4.7)}$$

Further, in each of these populations S_i, the evolution of the intracellular concentration of ribavirin phosphorylated analogs follows,

$$dC_i(t)/dt = k_p C_c(t) - k_d C_i(t); \quad C_i(t_i) = 0 \qquad \text{(S4.8)}$$

Equations (S4.6)-(S4.8) are a set of ordinary differential equations that are readily solved and yield the population dynamics of RBCs in HCV patients undergoing combination therapy.

The above solution technique based on the method of characteristics follows from an earlier technique employed to solve problems in chemical engineering [1].

SUPPLEMENTAL REFERENCE

1. Kumar S, Ramkrishna D (1997) On the solution of population balance equations by discretization--III. Nucleation, growth and aggregation of particles. Chem Eng Sci 52: 4659-4679.
