# Supplementary material for: Ribavirin-Induced Anemia in Hepatitis C Virus Patients Undergoing Combination Therapy
Source: PLoS Comput Biol. 2011 Feb 3;7(2):e1001072. doi: 10.1371/journal.pcbi.1001072 (PMC3033369; doi:10.1371/journal.pcbi.1001072)
Supplement: Text S5 — MATLAB program for solving model equations. (0.19 MB PDF) [file pcbi.1001072.s009.pdf]

MATLAB program for solving model equations

Below is the program we employ for integrating model equations (Eqs. (4)).

```
%**************************************************************************
function RBVanemia
%All quantities here are dimensionless.
%We nondimensionalize the number of erythrocytes with 2.5e13, Hb with 15 g/dL, volume
%with 5 L, concentration with 2 mM and time with 1 d.
%Parameter values are mentioned in Table 1.
global k kp dt N0 j Cpmax Cp ta kd C50 gamma Hb0 y0 D0 theta b Pmax D
%Output time points
time=0:1:28;
%Assign parameter values – symbols have the same meanings as in the manuscript
Hb0=14.4/15;
ta=5.4;
D0=0.00833;
%Nondimensional N=nondimensional Hb
N0=Hb0;
Cpmax=7.5/2000;
Pmax=0.0334;
b=7;
theta=(N0)*(Pmax/(D0*N0)-1)^(-1/b);
kp=65;
kd=0.5;
C50=0.2;
gamma=1;

len=length(time);
endtime=time(len);
dt=0.01;
k=(100+ceil(endtime/dt));

%Initialization of variable arrays (y(1:k) store Si and y(k+1:2k) store Ci)
y0(1:2*k)=0;
D(1:2*k)=D0;
y0(1)=N0;
no_cells(1)=sum(y0(1:k));
hb(1)=Hb0;
deltaHb(1)=0;
Cavg(1)=0;
Davg(1)=D0;
%Integration of model equations
j=1;
for t_out=2:1:len
```

```
   final=time(t_out);
   start=time(t_out-1);
   while start<final
       tspan=[start start+dt];
       j=round(start/dt)+1;
       Cp=Cpmax*(1-exp(-start/ta));
       [T,Y]=eul(@diff,tspan,y0,dt);
       fi_l=length(Y(:,1));
       for ee=1:2*k
           y0(ee)=Y(fi_l,ee);
       end;
       start=start+dt;
   end
   %Computation of output quantities
   no_cells(t_out)=sum(y0(1:k));
   sumCCp=0;
   sumD=0;
   for i=1:k
      sumCCp=sumCCp+y0(i)*(y0(k+i)+Cp);
      sumD=sumD+y0(i)*D(i);
   end;
   hb(t_out)=no_cells(t_out);
   deltaHb(t_out)=hb(t_out)-Hb0;
   Cavg(t_out)=sumCCp/no_cells(t_out);
   Davg(t_out)=sumD/no_cells(t_out);
 end;

%*************************************************************************
 %Differential equations (Eqs. (4) in the manuscript)
function  dy =diff(t,y)
global k kp theta b Pmax D0 Cp j kd C50 gamma D
dy = zeros(k*2,1);
total=sum(y(1:k));
P=Pmax*(theta.^b)/((total.^b)+(theta.^b));
%Time evolution of existing cell subpopulations.
for i=1:j
    D(i)=D0*(1+((y(i+k).^gamma)/(C50.^gamma)));
    dy(i)=-D(i)*y(i);
end;
%Time evolution of newly produced cells.
dy(j+1)=P-D(j+1)*y(j+1);
for i=k+1:j+1+k
%Time evolution of Ci
    dy(i)=kp*Cp-kd*y(i);
end
%*************************************************************************
```
