# Supplementary material for: Ribavirin-Induced Anemia in Hepatitis C Virus Patients Undergoing Combination Therapy
Source: PLoS Comput Biol. 2011 Feb 3;7(2):e1001072. doi: 10.1371/journal.pcbi.1001072 (PMC3033369; doi:10.1371/journal.pcbi.1001072)
Supplement: Text S6 — Validation of the solution methodology. (0.20 MB PDF) [file pcbi.1001072.s010.pdf]

Validation of the solution methodology

We test our solution methodology by comparison of the long-time ($t \rightarrow \infty$) steady state solution obtained by integrating Eq. (4) numerically and the analytical solution of Eq. (1) when $D(C)$=D_0. At steady state, i.e., when $\partial n / \partial t = 0$, Eq. (1) becomes

$$\frac{\partial}{\partial C}(Q(C, C_c)n(C)) = n(C)D_0 \qquad \text{(S6.1)}$$

Substituting $Q(C, C_c) = k_p C_c - k_d C$ and rearranging, Eq. (S6.1) yields the ordinary differential equation

$$\frac{\partial n(C)}{\partial C} = n(C)\frac{(D_0 - k_d)}{k_p C_p^{\max} - k_d C} \qquad \text{(S6.2)}$$

where we recognize that $C_c = C_p^{\max}$ at steady state. We integrate Eq. (S6.2) with the boundary condition that $n(0) = P(t \rightarrow \infty)/Q(0, C_c) = N_0 D_0 / k_p C_p^{\max}$, where the latter equality follows from the recognition that when $D(C)$=D_0, ribavirin accumulation does not induce anemia so that the RBC production rate is constant throughout at $P_0 = N_0 D_0$. The steady state solution is thus,

$$n(C) = \frac{N_0 D_0}{k_p C_p^{\max}}\left(1 - \frac{k_d C}{k_p C_p^{\max}}\right)^{\frac{D_0}{k_d} - 1} \qquad \text{(S6.3)}$$

which yields the cumulative distribution $m(C) = \int_0^C n(C)dC$ at steady state as

$$m(C) = N_0\left(1 - \left(1 - \frac{k_d C}{k_p C_p^{\max}}\right)^{\frac{D_0}{k_d}}\right) \qquad \text{(S6.4)}$$

$m(C)$ calculated using Eq. (S6.4) and obtained by solving Eq. (4) with $D(C)$=D_0 are in exact agreement (Fig. S3) and present a successful test of our solution methodology.
